# Acknowledgement to Reviewers of *Animals* in 2018

**DOI:** 10.3390/ani9010026

**Published:** 2019-01-14

**Authors:** Animals Editorial Office

**Affiliations:** MDPI, St. Alban-Anlage 66, 4052 Basel, Switzerland

Rigorous peer-review is the corner-stone of high-quality academic publishing. The editorial team greatly appreciates the reviewers who contributed their knowledge and expertise to the journal’s editorial process over the past 12 months. In 2018, a total of 246 papers were published in the journal, with a median time to first decision of 16 days and a median time to publication of 43 days. The editors would like to express their sincere gratitude to the following reviewers for their cooperation and dedication in 2018:

Abuelo, ÁngelLee, CarolineAdachi, IkumaLeiber, FlorianAdler, Steffen A.Lerner, HenrikAlbertini, MariangelaLesimple, ClémenceAllard, StephanieLewis, EvaAmendola, LuciaLin, ZhenguoAnastasiadou, MariaLiu, WanshengAndrews, ClareLiu, ZhiqianAparicio, Miguel A.Lloyd, JaniceAppleby, MichaelLópez Bote, Clemente JoséArakawa, HiroshiLopez-Bejar, ManelArney, DavidLorenzo Rodriguez, Jose ManuelArnott, GarethLovercamp, KyleArsenos, GeorgiosLukas, DieterAsher, LucyLulich, JodyAtes, SerkanLürzel, StephanieAveros, XavierLutz, CorrineAzarpajouh, SamanehLuzón Peña, MónicaBackus, Brittany L.MacKay, JillBadawi, MohamedMacLeod, MichaelBailey, HartfordMagalhães Sant’Ana, ManuelBaker, LivMahoney, Peter J.Baker, SandraMainau Brunso, EvaBandara, Rathnayaka AmilaMaiorano, GiuseppeBanko, PaulMakecha, RadhikaBaragli, PaoloMakowska, I. JoannaBarbieri, SaraMalashichev, YegorBarnard, ShanisMancini, SimoneBarwick, JamieMandanici, EmanueleBashaw, Meredith J.Manning, LouiseBasini, GiuseppinaManor, JuliaBaumans, VeraManteca, XavierBaxter, EmmaMaple, TerryBeausoleil, NgaioMapletoft, ReubenBeck, AlanMarcellin-Little, DenisBeerda, BonneMarchant-Forde, Jeremy N.Beggs, DavidMarechal, LaetitiaBenjamin, MadonnaMargerison, JeanBenson, EricMarini, DanilaBentosela, MarianaMaros, KatalinBernstein, MarkMarriott, Bernadette P.Bertoni, GiuseppeMarshall-Pescini, SarahBiase, FernandoMartin, JessicaBidarimath, MallikarjunMartin, VictoriaBionaz, MassimoMartinez-Pastor, FelipeBiscarini, FilippoMason, AlexBissonnette, NathalieMather, JenniferBjalobok, FaithMatthews, LindsayBjörkman, StefanMattioli, SimonaBland, IanMaurer, PatricBlatchford, Richard A.Mazzola, Silvia MichelaBoe-Hansen, GryMcBride, AnneBombail, VincentMccallum, HamishBonati, BeatriceMcCulloch, StevenBorecki, MichalMcGlone, JohnBoumans, Iris J.M.M.McGuire, BettyBovera, FulviaMcKeegan, DorothyBover-Arbos, PereMcLean, AmyBoyer Ontl, KellyMcLennan, KristaBoyle, LauraMeehan, Cheryl L.Brannick, Erin M.Meijboom, FranckBrook, RyanMejdell, CecilieBroom, DonaldMena, IgnacioBrown, HiekeMerchant, HamidBrown, SarahMerkies, KatrinaBrown, JenniferMeyer, Robert E.Buitenhuis, Albert JohannesMiguel-Pacheco, Giuliana G.Buller, HenryMilkov, AlexeiBuning, Tjard De CockMillemann, YvesBurghardt, PaulMiller, Stephen P.Burke, Joan M.Miron, JoshuahBushby, Philip A.Miskulin, MajaCabrita, Ana Rita JordãoMitchell, RobertCacciotto, CarlaMoate, Peter J.Cakebread, PeterMøller, Steen HenrikCalver, Michael C.Moons, ChristelCamerlink, IreneMordenti, AttilioCammack, JonathanMorgan, KathleenCampbell, DanaMornement, KateCampo, María Del MarMorris, Kevin N.Cantalejo, María J.Morsy, SalemCao, DeborahMorton, DavidCarella, PhilipMoya, DiegoCarney, Hazel C.Muboko, NeverCarragher, JohnMugnai, CeciliaCarter, AnneMullens, BradleyCartner, Samuel CorbinMüller, ArianeCarver, ScottMunoz, CarolinaCastaldo, LucianaMunsterhjelm, CamillaCastellini, CesareMurugesan, G. RajCaulfield, MalcolmMusk, Gabrielle C.Ceballos, Maria CamilaNagai, KouheiCeciliani, FabrizioNahid, Abdullah-AlCelaya, RafaelNam, Jin-WuCenci-Goga, Beniamino T.Nanni Costa, LeonardoChadwin, RobinNannoni, EleonoraClark, EmilyNapolitano, FabioClay, ZannaNg, ZenithsonClubb, RosNilon, Charles H.Collins, TeresaNishida, TakehiroComizzoli, PierreNorwood, F. BaileyConte, SabineNout-lomas, YvetteCoombe, JoNowicki, StacyCoppola, Crista L.Ogunade, IbukunCorino, CarloOikonomou, GeorgeCork, SusanO’Quin, JeanetteCorpa Arenas, Juan ManuelOrmandy, ElisabethCorti, ClaudiaOrozco-terWengel, PabloCosta, MarcioO’Shea, CormacCosta, AnnamariaOsthaus, BrittaCostall, AlanO’Sullivan, SiobhanCoulson, GraemeOviedeo, EdgarCoureaud, GérardOwen, HelenCove, MichaelOzella, LauraCozzi, AlessandroPacker, RowenaCrandell, KathleenPadalino, BarbaraCroft, David B.Pakozdy, AkosCronin, Katherine A.Palme, RupertCrowther, MathewPaneru, BamCzycholl, IrenaPapworth, SarahD’Occhio, MichaelPark, SimonDahlke, GarlandParker, ChristineDai, FrancescaPasicka, EdytaDaigle, Courtney L.Passantino, AnnamariaDalla Villa, PaoloPaterson, MandyDallaire, Jamie AhloyPatronek, GaryDalton, HillaryPauselli, MarianoDashper, KatePawley, MatDavis, Michael E.Peggs, KayDe Andrés Cara, Damián F.Peiretti, Pier GiorgioDe Briyne, NancyPellis, Sergio M.Delgado, MikelPeltoniemi, OlliDeMello, MargoPérez, ValentinDennis, IsabellePeterson, SamDescovich, KrisPieramati, CamilloDettori, Maria LuisaPino, Manuel M. Sánchez DelDeVries, TrevorPinotti, LucianoDi Giminiani, PierpaoloPiras, CristianDi Pasquale, JorgelinaPirgozliev, VasilDickman, ChristopherPoli, AlessandroDickson, CassandraPopova, MilkaDiggles, BenjaminPoulsen, Nina AagaardDiverio, SilvanaPound, PandoraDixon, LauraPourlis, ArisDodds, W. JeanPowell, DavidDolan, Emily D.Prescott, MarkDomisch, TimoPrunier, ArmelleDonnett, UriPulina, GiuseppeDonoghue, AnnieQuattrocchi, FedoraDorman, David C.Raedts, PieterDoughty, AmandaRagkos, AthanasiosDraper, ChrisRakhshandeh, AnooshDrigo, MicheleRandle, HayleyDudley, Emily SuzanneRanglack, Dustin H.Dukkipati, RaoRapi, StefanoDunner, SusanaRawlings, John MerrittDunston, EmmaRay, ParthaDuran, Sue HudsonReese, LauraDuranton, CharlotteReines, Brandon P.Dyson, SueReiss, MichaelDzidic, AlenRempel, LeaEastwood, CallumRenna, ManuelaEddison, JohnRiedstra, Bernd J.Edinboro, Charlotte H.Riemer, StefanieEdwards, SandraRiley, ChristopherEilam, DavidRivero Viera, Myriam JordanaEkenstedt, KariRobbins, JesseElmi, AlbertoRobertson, AndyElwood, Robert W.Robins, AndrewErickson, PeterRodenburg, BasEssler, JenniferRodriguez Sosa, Jose RFàbrega, EmmaRogers, Chris W.Fahey, GeorgeRollin, BernardFarnworth, MarkRommers, JorineFatjo, JaumeRoot-Gutteridge, HollyFaucitano, LuigiRoss, StephenFaulkes, ZenRossi, JohnFawcett, AnneRoth, Lina S.V.Faye, BernardRuggeri, PaoloFazio, FrancescoRząsa, AnnaFazio, EsterinaSakai, MasahiroFei, Andrew Chang-YoungSalak-Johnson, JaneenFerrante, ValentinaSalas, MarinaFiset, SylvainSalazar-Villanea, SergioFisher, MarkSaleri, RobertaFitzsimmons, CarolynSalzano, AngelaFleming, TrishSandøe, PeterFleming, SherrillSant’Ana, Manuel MagalhãesFlint, Hannah E.Scanes, Colin G.Forkman, BjörnSchiavone, AchilleForrest, RachelSchinckel, Allan PFoster, Derek M.Schjøll, AlexanderFranco, Nuno HenriqueSchmoelzl, SabineFreire, RafaelSchoster, AngelikaFriel, MarySchrade, SabineFriend, TedSchwarzer, AngelaFrye, ChristopherScofield, RoseFugazza, ClaudiaScollo, AnnalisaFujimori, KoScott, IanFumarco, LucaSearcy, WilliamFuß, RolandSeddon, Philip J.Gabler, NicholasSerpell, JamesGabriel, MouradSharafeldin, Tamer A.Gai, FrancescoSharp, TrudyGalligan, ThomasSheen, Shyn-ShinGallup, Gordon G.Shelton, DeliaGama, AdelinaSherwen, SallyGandy, Elizabeth A.Shevlin, HenryGarcés Narro, CarlosShil, NirajGarcia, MaximeSiegford, JaniceGarcía, ArleneSiettou, ChristinaGarstang, MichaelSimitzis, PanayiotisGates, CarolynSimojoki, HeliGazzano, AngeloSiniscalchi, MarcelloGebhardt-Henrich, SabineSinski, JenniferGeers, RonySlater, MargaretGeorg, HeikoSmall, AlisonGerritzen, MarienSmith, GrahamGetty, Kelly J.K.Smith, EdGibson, JohannaSnowdon, CharlesGiersberg, Mona FranziskaSparrey, JulianGill, RobinSpence, KelseyGingerich, EricSpengler Neff, AnetGonzalez-Rivas, PaulaSpörndly, EvaGootwine, ElishaSpricigo, Jose FelipeGordon, StuartStadig, Lisanne M.Gottdenker, NicoleStafleu, FransGouveia, KellyStafuzza, Nedenia BonvinoGramza, AshleyStanley, DraganaGrandin, TempleStear, MichaelGrant, RachelSteinwidder, AndreasGrau, AaronStella, JudithGreer, AlanStella, JudiGroße-Brinkhaus, ChristineStephan, WildeusGroves, PeterStephenson, Mitchell B.Ha, James C.Stilwell, GeorgeHabib, IhabStocco, CarlosHakkenberg, ChristopherSuchak, MaliniHalas, VeronikaSullivan, Megan LHall, KatieSuperchi, PaolaHan, Jong WonSuriyampola, PiyumikaHanlon, Alison J.Sutaria, DhruvitkumarHänninen, LauraSutherland, MhairiHansen, Axel KornerupTakahashi, Mizuki K.Harrison, DorotheaTallet, CélineHärtle, SonjaTaylor, PetaHartnack, SonjaTelesca, VitoHarvey, NaomiTeseo, SerafinoHässig, MichaelTheodoridis, AlexandrosHawkins, PennyTheuerkauf, JörnHazel, SusanThompson, JeffreyHeath, SebastianThomson, JackHedges, SimonThomson, JenniferHeiss, EgonThomson, DanHeleski, CamieThornber, PeterHendricks, Joan C.Thornton-Kurth, KaraHenriksen, Britt I.F.Tilbrook, AlanHerbut, PiotrTiplady, CatherineHernandez-Gomez, ObedTokuda, MasaharuHerring, Andy D.Toms, DerekHessle, AnnaTorremorell, MontserratHeymann, Eckhard W.Trabalza-Marinucci, MassimoHiby, EllyTrevarthen, AnnaHickman, DebraTreves, AdrienHill, JonathanTsukahara, TakamitsuHinton, Joseph W.Tufarelli, VincenzoHirase, ShotaroTurner, SimonHof, AnouschkaUpton, SarahHoffmann, GundulaUrfer, SilvanHofmeister, Erik K.Uwiera, RichardHolman, BenjaminVaarst, MetteHolst, Bodil StrömVan Eerdenburg, Frank J. C. M.Horback, KristinaVan Emon, MeganHoudebine, Louis-MarieVan Heezik, YolandaHoupt, Katherine AlbroVan Niekerk, TheaHowell, TiffaniVan Os, JenniferHuber, LudwigVan Staaveren, NienkeHughes, KateVan Uhm, DaanHume, Michael E.Van Winden, StevenHuntington, Gerald B.Vanni, FrancescoHutton, VickiVassalos, MichaelImumorin, Ikhide G.Vaughn, Mathew.A.Irvine, LeslieVentrella, DomenicoIson, Sarah H.Visscher, ChristianIwata, HisatakaVitale, AugustoJacob, RobinVon Essen, EricaJacobs, LeonieVon Keyserlingk, MarinaJaja, IshmaelVonk, JenniferJamieson, JenVyas, DiwakarJanakiraman, HarinarayananWallace, RyanJang, Young DalWallace, RobertJobling, MalcolmWallach, ArianJohnsson, MartinWallis, LisaJongman, EllenWalter, W. DavidKaburu, StefanoWang, Fun-InKang, Li-WeiWarwick, CliffordKang, SeongWashburn, SteveKasarda, RadovanWatson, AndreaKastelic, JohnWatters, JasonKaufmann, OttoWebster, JohnKedrowicz, April AnnWechsler, BeatKenéz, ÁkosWeisbjerg, MartinKiley-Worthington, MartheWells, DeborahKim, LeungWels, HarryKirkwood, RoyWeng, Hsin-YiKirnan, JeanWestman, MarkKjos, Nils PetterWeston, Michael AKleen, Joachim LübboWhite, GavinKleyheeg, ErikWhitehouse, JamieKloeze, HaroldWhiting, TerryKnight, PeterWickersham, TryonKokoszyński, DariuszWilburn, DamienKoliesnik, IevgenWilliams, JaneKong, ChangsuWilson, HerbKongara, KavithaWindisch, WilhelmKotani, TomoyaWindschnurer, InesKotrschal, KurtWoinarski, JohnKrawczel, Peter D.Wolfensohn, SarahKreisler, RachaelWoodworth, Jason C.Kröger, SusanWrege, PeterKues, Wilfried A.Würbel, HannoKumar, PraveenWynne, CliveKutschera, UlrichYau, YungLa Mendola, DiegoYonekura, ShinichiLadeira, CarinaYonezawa, TomohiroLameira, Adriano R.Yorzinski, JessicaLang, FionaYoung, RobertLaptikhovsky, VladimirZahan, MariusLasagna, EmilianoZaleski, HalinaLashley, VictoriaZhang, GuanshiLatorre, Juan DavidZhang, XiaohengLaurence, MichaelZhao, YangLaven, RichardZhong, LihengLeach, MatthewZienius, DainiusLee, Kyung-Woo


